# Potential Targets of Natural Products for Improving Cardiac Ischemic Injury: The Role of Nrf2 Signaling Transduction

**DOI:** 10.3390/molecules29092005

**Published:** 2024-04-26

**Authors:** Haixia Wang, Juanjuan Han, Gorbachev Dmitrii, Xin-an Zhang

**Affiliations:** 1 College of Exercise and Health, Shenyang Sport University, Shenyang 110102, China; whx1415@163.com (H.W.); hanhan9210@163.com (J.H.); 2 Department of Sport Rehabilitation, Shanghai University of Sport, Shanghai 200438, China; 3 General Hygiene Department, Samara State Medical University, Samara 443000, Russia; d.o.gorbachev@samsmu.ru

**Keywords:** Nrf2, Keap1/Nrf2/HO-1, natural products, myocardial ischemia, myocardial ischemia-reperfusion, myocardial infarction

## Abstract

Myocardial ischemia is the leading cause of health loss from cardiovascular disease worldwide. Myocardial ischemia and hypoxia during exercise trigger the risk of sudden exercise death which, in severe cases, will further lead to myocardial infarction. The Nrf2 transcription factor is an important antioxidant regulator that is extensively engaged in biological processes such as oxidative stress, inflammatory response, apoptosis, and mitochondrial malfunction. It has a significant role in the prevention and treatment of several cardiovascular illnesses, since it can control not only the expression of several antioxidant genes, but also the target genes of associated pathological processes. Therefore, targeting Nrf2 will have great potential in the treatment of myocardial ischemic injury. Natural products are widely used to treat myocardial ischemic diseases because of their few side effects. A large number of studies have shown that the Nrf2 transcription factor can be used as an important way for natural products to alleviate myocardial ischemia. However, the specific role and related mechanism of Nrf2 in mediating natural products in the treatment of myocardial ischemia is still unclear. Therefore, this review combs the key role and possible mechanism of Nrf2 in myocardial ischemic injury, and emphatically summarizes the significant role of natural products in treating myocardial ischemic symptoms, thus providing a broad foundation for clinical transformation.

## 1. Introduction

Cardiovascular diseases (CVDs) are the leading cause of premature death and chronic disability worldwide, and the occurrence of cardiovascular events has become an increasingly serious public health issue [1]. Epidemiology shows that the prevalence of CVD has increased from 271 million in 1990 to 523 million in 2019 [1,2]. Among them, ischemic heart disease is the main cause of cardiovascular health loss worldwide and in various regions around the world. The incidence rate in China is increasing year by year, mainly affecting middle-aged and elderly people, and gradually becoming younger. In severe cases, it will further lead to myocardial infarction (MI) [3,4]. Percutaneous coronary intervention, which is commonly used in clinics, has achieved timely myocardial reperfusion and is the most effective treatment [5,6]. However, once the blood supply is restored, the original ischemic myocardium will suffer from cellular metabolic dysfunction, and even irreversible damage to the tissue structure, namely myocardial ischemia-reperfusion injury (MIRI) [7]. Eventually, it will induce cardiomyocyte injury, apoptosis, inflammation, microvascular endothelial injury and no reflux phenomenon, which will directly affect the clinical efficacy of patients [8]. Given the severity and complexity of these diseases, it is crucial to seek new therapeutic targets to improve myocardial ischemic injury.

Nuclear factor E2-related factor 2 (Nrf2) belongs to the Cap ‘n’ Collar (CNC) family, and is encoded by the *NFE2L2* gene [9,10]. Due to its crucial role in regulating the expression of antioxidant and detoxifying enzymes, Nrf2 has become a widely studied hotspot [11,12]. Under non-stress conditions, Nrf2 is degraded by binding to Kelch-like ECH-associated protein 1 (Keap1), an adaptor protein for E3 ubiquitin ligases. In response to stimuli such as oxidative stress, Nrf2 rapidly accumulates and translocates to the nucleus, where it forms a heterodimer with one of the small musculoaponeurotic fibrosarcoma proteins (sMaf) [10]. Nrf2 binds to the regulatory region of the target gene and upregulates its transcription. As one of the coordinators of exogenous and oxidative stress, Nrf2 mainly plays a role in organs with strong metabolism, such as cardiovascular system, liver and skeletal muscle, and induces the expression of a series of cell protection gene products to protect cells from oxidative stress [13,14,15]. Evidence shows that the function of Nrf2 mainly focuses on the regulation of redox ability, including inflammation, autophagy, metabolism, antioxidation, protein homeostasis and ferroptosis, so as to support it as a protective factor of myocardial ischemia [16,17,18,19]. The regulatory system of Nrf2 activity has become a very attractive drug target [20].

Because of their significant biological activity, varied chemical structures, and low side effects, natural products (NPs) are widely employed in the treatment of many cardiac ailments [21,22]. Furthermore, they are an important source of bioactive compounds for the creation of new drugs [23]. Evidence exists to support the importance of dietary therapy and medicine in the treatment of chronic ischemic heart disease. Cardiac function can be markedly improved by supplementing NPs-derived nutritional substances or by utilizing them as a pretreatment during myocardial ischemia [24]. More and more NPs are regulating Nrf2 related pathways to enhance endogenous antioxidant defense, thereby reducing myocardial cell apoptosis, inflammation, oxidative stress, fibrosis, and ferroptosis, which is expected to alleviate MI [25,26]. Unfortunately, there is no relevant report about the involvement of Nrf2 in the improvement of MI by NPs. Therefore, this review focuses on elucidating the relevant mechanisms of different types of NPs delaying the progression of myocardial ischemia through Nrf2, emphasizing the potential of NPs in treating myocardial injury. We hope that this review can promote new ideas for developing natural products targeting Nrf2 as anti-myocardial ischemia drugs.

## 2. Nrf2 Overview

### 2.1. Molecular Structure of Nrf2

**Figure 1:**
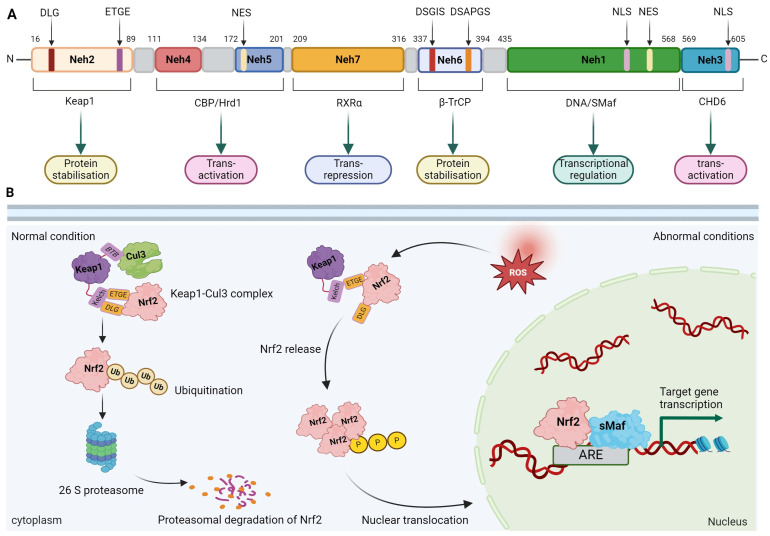
Molecular structure and signaling pathway activation mechanism of Nrf2. (**A**): Nrf2 molecular structure; (**B**): Nrf2 signaling pathway activation mechanism (created with BioRender.com, accessed on 10 March 2024).

The basic-region leucine zipper (bZIP) transcription factor CNC family encoded by the *NFE2L2* gene includes nuclear factor erythroid-derived 2 and NFE2-related factors Nrf1, Nrf2 and Nrf3 [11,27]. Nrf2 has become the most important member of this family because of its important role in preventing oxidative stress and electrophilic stress [28]. It contains 605 amino acids and seven highly conserved functional domains, known as Nrf2-ECH homology domains (Neh1-Neh7) [29]. Neh1 contains a bZIP motif that regulates DNA binding [30], which forms heterodimers with sMaf K, G and F [31], and binds to antioxidant response elements or electrophilic response elements (ARE/EpRE) in the nucleus, thereby regulating the transcription of various antioxidant enzymes, phase II detoxification enzymes, ATP-binding cassette (ABC) transporters and other stress response proteins [32,33]. In addition, nuclear localization signal (NLS) and nuclear export sequence (NES) exist in this region to regulate the nuclear translocation of Nrf2 [34]. The domain located at the N-terminus of Nrf2 is called Neh2, which is the most important regulatory domain in Nrf2 and is mainly responsible for regulating the stability of Nrf2. The ETGE motif and DLG motif contained in this domain interact with the Kelch domain of its negative regulator Keap1, and subsequently inhibit Nrf2 by regulating Keap1-dependent ubiquitination and proteasomal degradation [35,36]. The Neh3 domain is located at the C-terminus of Nrf2 and binds to CHD6, a member of the helicase DNA binding protein family [37], which can transactivate dependent genes [38,39]. Neh4 and Neh5 interact with cAMP response element binding protein (CREB)-binding protein (CBP) with histone acetyltransferase activity to support transcriptional activation [40,41]. Neh6 and Neh7 domains are negative regulators of Nrf2 activity. The Neh6 domain contains two redox-independent degrons, DSGIS and DSAPGS, for recognizing ubiquitin ligase adaptors containing β-transducing repeat-containing protein (β-TrCP), involved in Nrf2 Keap1 independent ubiquitination [42,43]. The seventh Neh7 domain promotes Nrf2 and its repressor retinoic X receptor alpha (RXRα) binding to inhibit Nrf2 target gene transcription [44] (Figure 1).

### 2.2. Regulation of Nrf2 Activity

Nrf2 is mainly negatively regulated by Keap1, which is an oxidation–reduction sensitive E3 ubiquitin ligase substrate junction [45,46]. Keap1 belongs to the BTB Kelch protein family, and is a cysteine rich protein [47]. It can be divided into five domains: an N-terminal region (NTR), a BTB domain, a central interference region (IVR) with NES mediated Keap1 cytoplasmic localization, six Kelch repeat sequences, and a C-terminal region (CTR) [45,48,49]. Among them, the BTB domain promotes the binding with cullin (Cul)3 E3 ligase, forming the Keap1-Cul3-ring box 1(RBX1) E3 ubiquitin ligase complex, which is necessary for Keap1 homologous dimerization [50]. The Kelch domain located at its C-terminus can interact with the ETGE and DLG motifs in the Neh2 domain of Nrf2, serving as an adapter for the Cul3/RBX1 E3 ubiquitin ligase complex, binding Nrf2 in dimeric form and promoting its ubiquitination [51,52,53]. IVR is located between the BTB and Kelch/DGR domains and contains cysteine residues, which can regulate the activity of Keap1 [48,54]. These three main structural domains play an important role in maintaining stability between Nrf2 and Keap2.

Under normal physiological conditions, Nrf2 is sequestered in the cytoplasm by Keap1. The BTB structural domain of Keap1 forms a Keap1–Cul3–RBX1 E3 ligase complex with Cul3 ligase, and interactions between the Kelch structural domain of Keap1 and the ETGE and DLG motifs on the Neh2 structural domain of Nrf2 localize Nrf2 in the Keap1 in a complex that ultimately leads to the degradation of Nrf2 by the 26S proteasome and proteasomal ubiquitination [31,55]. Under oxidative stress or other stimulatory conditions, the Kelch structural domain of Keap1 is altered. Since the binding affinity of the ETGE motif of Nrf2 is higher than that with the DLG motif, Nrf2 can still attach to Keap1 via the ETGE motif [35,56]. However, the low binding affinity DLG motif dissociates from Keap1, thus preventing the ubiquitination and degradation of Nrf2 [56]. At this point, Nrf2 is no longer a target for degradation by Keap1 molecules, and Nrf2 is released from the Keap1–Cul3–RBX1 complex and translocated to the nucleus. In the nucleus, Nrf2 dimerizes with members of the sMaf family of proteins, which activates the transcription of gene cascades containing AREs or EpREs in the promoter region, thereby increasing the transcriptional activity of a range of antioxidant enzymes, such as HO-1, SOD, NQO1, CAT, GSH, and GPX [57,58]. The Nrf2-sMaf protein heterodimer regulates the transcription of ARE-containing target genes through the recruitment of transcriptional coactivators which, in turn, are involved in a variety of cellular processes, mainly involving the regulation of redox homeostasis, autophagy, apoptosis, iron-homeostasis, DNA repair, transcriptional regulation, phase I, II and III drug/metabolism, carbohydrate and lipid metabolism, and proteasome assembly [58,59]. By inducing the expression of these genes, Nrf2 is able to enhance cellular defense processes against damage from external stimuli. This cellular defense pathway is known as the Keap1–Nrf2 system (Figure 1).

## 3. Regulation of Nrf2 on Myocardial Ischemia

### 3.1. Nrf2/HO-1

Heme oxygenase-1 (HO-1), as an important target downstream of Nrf2, can catalyze the degradation of heme into biliverdin, free iron and CO, thus playing antioxidant, anti-inflammatory, anti-apoptotic and antithrombotic roles [59]. Nrf2/HO-1 plays a crucial role in combating various oxidative stress responses and cardiac remodeling after MI. When cardiomyocytes are attacked by a large number of reactive oxygen species (ROS), Nrf2 rapidly translocates to the nucleus, which activates the expression of antioxidant enzyme HO-1 by binding to the promoter region [60]. HO-1 activation can inhibit MIRI-induced inflammatory factor production during cardiopulmonary bypass and inhibit NF-κB and activator protein (AP)-1 translocation to reduce cardiomyocyte apoptosis [61], while pre-injection of HO-1 activator can significantly reduce MI infarct size and cardiomyocyte apoptosis [62]. HO-1 decomposes heme to produce CO, which can promote the proliferation of vascular endothelial cells and protect cardiomyocytes from oxidative stress by inhibiting L-type Ca^2+^ channels and T-type Ca^2+^ channels [63]. Relevant evidence shows that Nrf2/HO-1 can treat MI by reducing the inflammatory response, apoptosis and oxidative stress.

### 3.2. AMPK/GSK-3β/Nrf2

AMP-activated protein kinase (AMPK) and glycogen synthase kinase-3β (GSK-3β) play a key role in protecting cells from ischemic injury, and also affects glucose uptake and metabolism during MI [64,65]. Activating the AMPK/GSK-3β signaling pathway can increase the nuclear translocation of Nrf2, upregulate the transcription of HO-1, solute carrier family 7 member 11 (SLC7A11) and glutathione peroxidase 4 (GPX4), and reduce the expression of caspase 3 which, in turn, can combat oxidative stress, ferroptosis and cell apoptosis in myocardial ischemia/reperfusion (I/R) [66,67]. In addition, upregulation of the sirtuin family member sirtuin-3 (Sirt3) can enhance AMPK activity, thereby inhibiting ROS accumulation and myocardial apoptosis, while alleviating inflammatory response and improving myocardial I/R injury [68], but the specific mechanism of its downstream has not yet been elucidated. In summary, activation of AMPK/GSK-3β/Nrf2 signaling pathway protects the heart from I/R-induced oxidative stress, thereby alleviating cardiac dysfunction and injury.

### 3.3. PI3K/Akt/Nrf2

Phosphoinositide 3-kinase (PI3K) and its downstream target protein kinase B (Akt) have been identified as the key mechanisms of the occurrence, progression and treatment of MI. Induction of PI3K-Akt can also prevent MIRI by inhibiting mitochondrial permeability transition pore opening [69]. Similarly, inhibition of Phosphatase and tensin homolog (PTEN, a negative regulator of PI3K/Akt) can reduce the expression of caspase-3, caspase-7, and caspase-9, while increasing the expression of B cell lymphoma-2 (Bcl-2) in cardiomyocytes of MI mice, ultimately enhancing the protective effect of mice against ischemic injury after MI and reducing inflammation and myocardial remodeling [70]. Studies have shown that PI3K/Akt/Nrf2 pathway activation can upregulate Bcl-2 expression and down regulate cleaved caspase-3 and Bax, thereby alleviating oxidative stress and cardiomyocyte apoptosis and playing an anti-MI role [71]. PI3K/Akt dependent signal transduction pathway regulates Nrf2 transcriptional activity. Activating PI3K/Akt signal is accompanied by the increase of nuclear Nrf2 and HO-1, which promotes cardiac recovery after MIRI [72]. Conversely, Nrf2 nuclear translocation was abolished using PI3K inhibitors [73]. In addition, GSK-3β is a downstream molecule of Akt, which can phosphorylate GSK-3β and keep the enzyme inactive, which in turn enhances the activation of Nrf2 under I/R conditions [74]. It can be seen that PI3K/Akt/Nrf2 is an important mechanism to prevent cardiac ischemic injury.

### 3.4. NF-κB/p65/Nrf2

The activation of the NF-κB signaling pathway is essential for the pathogenesis of cardiomyocyte injury. NF-κB p65 synergizes with the coactivator HDAC3 to directly inhibit the activity of Nrf2 through deacetylation, thereby promoting I/R-induced cardiomyocyte necrosis [75]. Inactivation of NF-κB signaling can inhibit the release of inflammatory cytokines, reduce cardiomyocyte apoptosis and interstitial fibrosis, reduce the susceptibility to ventricular arrhythmias, and improve cardiac function [76], which revealed that the pathogenic role of NF-κB in cardiac ischemic injury and pathological remodeling [77]. Conversely, the Nrf2 pathway can reduce the activity level of NF-κB by increasing the levels of antioxidants and cytoprotective enzymes, preventing IκB-α (NF-κB inhibitor) degradation, thereby inhibiting NF-κB mediated transcription [78]. Therefore, inhibition by targeting NF-κB p65 activation of Nrf2 may be an important strategy to alleviate MI. However, it is necessary to further clarify other possible molecular crosstalk mechanisms, such as Sirt2 and Sirt6 [79,80].

### 3.5. Sirt1/Nrf2

Sirt1 is the most widely studied longevity factor among the seven members of the sirtuins family. Studies have observed that Sirt1 activation not only reduces cardiomyocyte apoptosis induced in diabetic cardiomyopathy models [81], but also inhibits oxidative stress in MIRI [82]. The protective effect conferred by Sirt1 on the heart involves the endogenous antioxidant system of Nrf2. Sirt1 mediates protein activity through the deacetylation of lysine residues, and its upregulation mediates Nrf2 deacetylation and increases the nucleocytoplasmic localization and transcriptional activity of Nrf2, while promoting the binding of Nrf2 to its DNA response element ARE, thereby reducing H9c2 cardiomyocyte apoptosis and oxidative stress and improving cardiac dysfunction [82]. However, Sirt1 specific inhibitors have largely blocked the protective effect of Nrf2 on MI in vitro and vivo [83]. However, Nrf2 siRNA has little effect on the expression and activity of Sirt1 [84]. Sirt1/Nrf2 signaling can also regulate I/R-induced endoplasmic reticulum stress and participate in the disruption of the cardiac microvascular endothelial barrier triggered by oxygen-glucose deprivation/reoxygenation (OGD/R) [85]. In conclusion, activating Sirt1/Nrf2 signaling pathway is a potential target to improve MI injury.

### 3.6. MAPK/ERK/Nrf2

The mitogen-activated protein kinase (MAPK) signaling pathway consists of c-Jun N-terminal kinases (JNKs), p38 mitogen activated protein kinase (p38) and extracellular signal regulated kinase-1/2 (ERK1/2). Among them, MAPK/ERK pathway exerts a cardioprotective effect on MIRI [86]. Phosphorylation of ERK1/2 induces the activation of Nrf2 which, in turn, upregulates HO-1 expression [87]. Notably, the removal of Bach1 (HO-1 transcriptional repressor) from the nucleus in an ERK1/2-dependent manner can change the time difference between NAD(P)H quinone dehydrogenase 1 (NQO1) and HO-1 increase after myocardial injury treatment [88]. However, the activation of MAPK signaling pathway does not always exert a protective effect on MIRI. Studies have found that activating p38 MAPK/JNK pathway inhibits cardiomyocyte viability and promotes apoptosis to aggravate MIRI [89,90]. The specific mechanism of p38 MAPK/JNK pathway regulating Nrf2 needs further elucidation. In conclusion, existing studies have shown that MAPK/JNK mediated activation of Nrf2 signaling partially contributes to cardioprotection.

**Figure 2:**
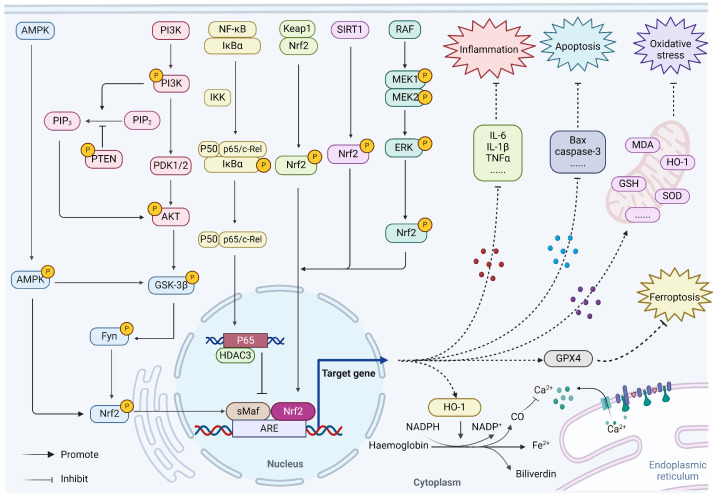
Regulation mechanism based on Nrf2 signal pathway. (Created with BioRender.com, accessed on 10 March 2024).

In conclusion, HO-1, AMPK, PI3K/Akt, NF-κB, Sirt1 and MAPK signaling pathways based on Nrf2-mediated signal play an important protective role against MI injury, but the crosstalk between multiple mechanisms needs further study (Figure 2).

## 4. Natural Products Target Nrf2 to Improve Myocardial Ischemia Injury

NPs have long been used to prevent and treat various diseases, and have become the main source of new drug research and development because of their unique active framework, active groups and excellent biological activities. According to their structural types, they can be divided into terpenoids, flavonoids, phenols, polysaccharides and glycosides, steroids, alkaloids, phenylpropane, quinones, etc. They rely on their pharmacological activities to protect the heart, such as enhancing cardiac remodeling, improving MI, relieving MI, and protecting hypoxia/reperfusion-induced myocardial apoptosis (Table 1).

### 4.1. Terpenoids

Andrographolide (Andr) is a labdane diterpenoid derived from the natural plant *Andrographis paniculata* [134,135]. It is widely used in the treatment of various diseases because of its antibacterial, anti-inflammatory, antioxidant and immunomodulatory effects. Andrographolide was found to inhibit expression of the inflammatory cytokine tumor necrosis factor-α (TNF-α), interleukin-1β (IL-1β), interleukin-6 (IL-6) and monocyte chemoattractant protein-1 (MCP-1), and NF-κB signaling pathway (p-IκBα and p-p65) activation, alleviating post MI inflammation. In addition to its anti-inflammatory activity, it also upregulates the levels of superoxide dismutase (SOD) 2, NQO1 and glutathione peroxidase (GPX) by activating Nrf2/HO-1 signaling, while reducing the transcription of p67 phox, Gp91 and NADPH oxidase 4 (NOX4), thereby inhibiting oxidative stress after myocardial infarction [91].

Panaxatriol saponin (PTS) isolated from ginseng can improve H_2_O_2_-induced cardiomyocyte redox homeostasis disorder and cardiomyocyte apoptosis, as shown by increased expression of SOD1, SOD2 and HO-1, and decreased expression of cleaved caspase-3, cleaved PARP-1, Bax, Cyt-c and ROS [92]. Panaxatriol binds to the Kelch domain of Keap1 protein, directly competes and restricts the binding site of Nrf2 and Keap1, destroys the negative regulation of Nrf2 by Keap1, and then prevents the ubiquitination and degradation of Nrf2. In vivo experiments found that it reduced the myocardial infarct size of I/R rats by targeting Keap1/Nrf2, and attenuated the expression of myocardial injury markers (MB, cTn-T, CK, LDH and CK-MB) [92].

Ginsenosides isolated from Panax notoginseng and ginseng include ginsenoside Rd (GsRd), ginsenoside Rh2 (GRh2) and ginsenoside Rbs [136]. GsRd can restore the impaired cardiac function caused by myocardial I/R injury in rats, reduce myocardial infarct size, and reduce serum CK, lactate dehydrogenase (LDH) and troponin (cTnI) levels by activating Nrf2/HO-1 signaling [93]. GRh2 increased the protein levels of nod-like receptor protein 3 (NLRP3), apoptosis-associated speck-like protein containing a CARD (ASC) and caspase-1 induced by myocardial I/R and hypoxia/reoxygenation (H/R), but increased the expression of Nrf2 and HO-1 [94]. This indicates that Nrf2/HO-1/NLRP3 signaling pathway may mediate GRh2 to protect the heart from oxidative stress and inflammatory response. However, the inevitable link between the two still needs further elucidation. Ginsenoside Rb2 is also considered to be beneficial for cardiovascular diseases [137]. Inactivation of the Nrf2/HO-1 signaling pathway alleviates the effects of ginsenoside Rb2 in promoting proliferation and inhibiting oxidative stress and apoptosis in H9c2 cells [95]. However, the specific protective mechanism of ginsenoside Rb2 mediated Nrf2/HO-1 signaling pathway on the heart needs to be further elucidated through in vivo experiments.

Triptolide (TPL) is a diterpene tricyclic oxide isolated from the traditional Chinese medicine plant Tripterygium wilfordii Hook F [138]. It can reduce the expression of TNF-α, IL-1β, IL-6 and malondialdehyde (MDA), and enhance the activities of antioxidants SOD, GPX and glutathione (GSH) in myocardial tissue of I/R rats by enhancing the nuclear accumulation of Nrf2 and the activity of its downstream target HO-1 [96]. In contrast, a blockade of the Nrf2/HO-1 signaling using zinc protoporphyrin-IX led to an attenuation of TPL-mediated cardiac protection [96].

Betulinic acid (BA) is a pentacyclic triterpenoid that is abundantly found in birch [139]. Studies have shown that BA protects H9c2 cells from oxidative stress and apoptosis caused by myocardial ischemia/reperfusion injury (I/RI), which is mediated by enhancing the activation of Nrf2/HO-1 pathway and inhibiting the activation of p38 and JNK pathways [97].

Maslinic acid (MA) belongs to natural pentacyclic triterpenoids and has good anti-inflammatory and anticancer properties [140]. It increases the activity of GSH and SOD by promoting the nuclear expression of Nrf2 to reduce the generation of ROS in a dose-dependent manner, alleviating MIRI and H_2_O_2_-induced oxidative stress in H9c2 cells. The above process can be reversed by using ML385 (Nrf2 inhibitor). In addition, NF-κB signaling pathway is also involved, but it is inhibited by MA [98].

The ent-kauranoid diterpenoid glaucocalxin A (GLA) has been proved to have a variety of pharmacological effects such as antioxidant, antifibrotic and immune regulation [141]. GLA inhibited ROS production and the activities of pro-apoptotic proteins Bax and caspase-3, and increased the expression of antioxidant enzymes (SOD and GSH-Px) and anti-apoptotic protein Bcl-2. Additionally, it can also significantly induce the expression levels of p-Akt, nuclear Nrf2 and HO-1, thereby preventing H/R-stimulated cell oxidative damage and apoptosis [99].

Costunolide (Cos) is a sesquiterpene lactone mainly found in the traditional Chinese medicinal herb Saussurea lappa, and its protective effect on the heart is gradually being discovered [142]. In vivo and in vitro studies confirmed that Cos significantly reduced ROS levels, increased the expression of antioxidant proteins (HO-1 and NQO1), and decreased Bax/Bcl-2 ratio, thereby improving I/R-induced cardiomyocyte apoptosis. When Nrf2 was silenced, the protective effect of Cos on H9c2 cells was weakened. Further study found that Cos significantly enhanced the dissociation of Keap1/Nrf2 complex, which is similar to the mechanism of PTS in protecting against myocardial injury [100].

Lutein (LU) is a major carotenoid derived from flower plants, vegetables, fruits and eggs [143]. Commercial antioxidant mixtures rich in LU protect the myocardium from I/R injury [144]. In the isoproterenol (ISO)-induced MI rat model, rats orally administered LU showed MI size, lipid peroxidation product MDA, cardiac diagnostic marker enzymes (cTnT, CK-MB and LDH), inflammatory factors (IL-1β, TNF-α, NF-κB p65 and IL-6) and apoptosis related markers (caspase-3 and caspase-9) were significantly decreased, while the activities of cardiac antioxidants SOD and catalase (CAT) were increased. In addition, the nuclear expression of Nrf2 was further upregulated by LU treatment, and the expression of HO-1 was also upregulated [101].

### 4.2. Flavonoids

Hesperetin (HESP), a member of the flavanone subclass of flavonoids, has a good curative effect on cardiovascular disease [145]. By activating Sirt1/Nrf2 pathway, it restored CK and LDH levels in serum, reduced Ca^2+^ concentration in mouse cardiomyocytes, and attenuated ISO induced the contents of MDA, IL-6, TNF-α,Bax and caspase-3 increased and the activities of SOD, CAT, GSH and Bcl-2 decreased [102]. In addition, HESP can also reduce cardiac injury, oxidative stress, apoptosis and Ca^2+^ influx of L-type Ca^2+^ channels caused by CoCl_2_ mimicking hypoxia, and protect myocardial injury induced by ischemia and hypoxia [146].

Baicalin (BI) is the main bioactive flavone derived from the medicine Radix Scutellariae, which has a variety of pharmacological activities, including anti-inflammatory, antioxidant and anti-apoptotic [147]. BI attenuates cardiomyocyte apoptosis and inflammatory factor infiltration in I/R model rats not only through the JAK/STAT pathway, but also involves the Nrf2/HO-1 pathway [148]. Silencing of hypoxia-inducible factor 1 alpha (HIF1α) changed the effect of baicalin on promoting H9c2 cell viability and inhibiting apoptosis. BI treatment not only further increased HIF1α and Bcl-2/adenovirus E1B interacting protein 3 (BNIP3) in hypoxia-induced H9c2 cells, which also promoted the expression of Nrf2 and HO-1. Interestingly, silencing Nrf2 reversed the protective effect of BI on myocardial injury and significantly reduced HIF1α [103]. This revealed that the mechanism of BI improving MI injury relies on activating Nrf2/HO-1-mediated HIF1α/BNIP3 pathway.

Pinocembrin (PCB) is a natural flavonoid found in propolis [149], which plays an anti-inflammatory, antioxidation and anticancer role. It improves cardiac function and remodeling in post-infarct heart failure (PIHF) by activating the Nrf2/HO-1 pathway, and attenuates ISO-induced ROS accumulation [105]. PCB also inhibits the expression of pyroptosis related factors NLRP3, ASC, cleaved caspase-1 and gasdermin D-N-terminal domain (GSDMD-N) by activating the Nrf2/Sirt3 signaling pathway, thereby reducing doxorubicin (DOX)-induced pyroptosis of cardiomyocytes and protecting the heart from cardiotoxicity [104].

Icariin (ICA) is a flavonol glycoside extracted from Epimedii Herba, which is beneficial for cardiovascular and neurological diseases [150,151]. Research has found that ICA alleviates H/R-induced ferroptosis and oxidative stress in cardiomyocytes by activating the Nrf2/HO-1 signaling pathway, manifested by increased cell viability and GPX4 levels while decreasing Fe^2+^, LDH and acyl-CoA synthetase long-chain family member 4 (ACSL4) levels [106].

Wogonoside (WG) is a flavonoid compound derived from the roots of the traditional Chinese medicine plant *Scutellaria baicalensis*. Previous studies have reported that active compounds in *Scutellaria baicalensis* can exert anti-inflammatory and immunomodulatory effects by affecting various signaling pathways, such as Nrf2, PPAR, MAPK, Akt, NF-κB and JAK/STAT signaling pathways and Toll-like receptors [152]. In a mouse model of myocardial I/R injury established in the left anterior descending coronary artery (LAD), after seven consecutive days of administration of 20 and 40 mg/kg WG to mice, it was found that abnormal structural recovery, apoptosis, myocardial fibrosis and inflammatory response of myocardial cells were significantly reduced. Importantly, WG exerts this effect by increasing the nuclear expression of Nrf2 and its downstream genes (HO-1 and NQO1), thereby delaying myocardial cell damage [107].

Visnagin (VIS) is an active compound that has been found to inhibit NF-κB and produces anti-inflammatory effects [153]. It upregulates myocardial Nrf2, HO-1, Bcl-2, and PPARγ in a dose-dependent manner in poisoned rats, and reduces the expression of apoptosis and pro-inflammatory cytokines such as Bax, caspases, ROS, MDA, NF-κB p65 can improve myocardial damage [108]. Therefore, the antioxidant, anti-inflammatory and anti-apoptotic effects of VIS in the heart may be related to the stimulation of the activation of Nrf2/HO-1 and PPAR-γ.

Isoliquiritigenin (ISL) is a chalcone type flavonoid derived from licorice, and its therapeutic effect has been confirmed in a variety of diseases [154]. In the mouse model of acute MI, the antioxidant effect of ISL was dependent on the activation of Nrf2/HO-1 signaling pathway, while attenuating inflammatory factors and chemokines (IL-6, IL-1β, TNF α, MIP1α and MIP2) through the inhibition of NF-κB signaling. Notably, the expression of p-IKKα/β, p-p65 and p-IκBα can increase in the NF-κB signaling pathway after inhibiting Nrf2, which indicates that Nrf2 can affect NF-κB signaling pathway activity and attenuate the inflammatory response [109].

### 4.3. Phenols

Salvianolic acid B (Sal B), a water-soluble compound extracted from the root of *Salvia miltiorrhiza*, has anti-atherosclerotic pharmacological effects [155]. It was found that the transcription of Nrf2 was activated in rats with MI, which was the feedback response of Nrf2 to oxidative stress. Sal B further activated the nuclear expression of Nrf2 and upregulated the levels of Nrf2 target genes HO-1, XCT, GPX4, Fpnl and Fthl in a dose-dependent manner. After knocking down Nrf2 in vivo, it was observed that the release of iron ions increased, and there was also a large accumulation of lipid peroxidation products, and Sal B treatment could not upregulate the expression of Nrf2 target genes [110]. In conclusion, Sal B’s protection against myocardial infarction by inhibiting ferroptosis is dependent on the activation of Nrf2 signaling pathway.

Lithospermic acid (LA) is also the main phenolic acid compound derived from *Salvia miltiorrhiza*, which has been proved to be used in the treatment of coronary heart disease angina [156,157]. As an oxidase inhibitor, LA can reduce the content of oxidative factors such as NOX4, p67 phox and GP91 in MIRI and promote the expression of antioxidant genes (GPX, SOD2 and NQO1) [111]. Studies have reported that AMPK triggered the phosphorylation of Nrf2 and promoted the transactivation of antioxidant genes [158]. Zhang confirmed through in vitro and in vivo studies that LA promotes the activation and phosphorylation of AMPKα, further leading to the nuclear translocation of Nrf2 in MIRI and activates the Nrf2/HO-1 pathway [111]. The phosphorylation of AMPKα seems to be a key factor in LA mediated activation of Nrf2/HO-1 signaling to ameliorate MIRI.

Kazinol B (KB) is a natural isoprenylated flavan, which is enriched in the root bark of *Broussonetia kazinoki* Sieb and has effective antioxidant and anti-inflammatory properties [159]. It not only significantly promoted Nrf2 nuclear accumulation and increased ARE promoter activity and HO-1 levels, but also upregulated phosphorylation of Akt and AMPKα in H/R-induced H9c2 cells. In addition, Akt and AMPK specific inhibitors abolished Nrf2 nuclear translocation [112]. The above shows that KB is expected to become a therapeutic drug for ischemic heart disease by affecting the phosphorylation level of AKT/AMPK and regulating Nrf2/ARE/HO-1 signaling.

Paeonol (Pae) is a phenolic acid compound purified from the root of *Paeonia lactiflora* Pall. In exploring the anti-apoptotic and antioxidant mechanisms of Pae and danshensu combination (PDSS) in the treatment of ischemic heart disease (IHD), it was found that PDSS not only significantly reduced the histopathological changes in rat myocardial tissue sections induced by ISO, but also decreased ROS, Bax, thiobarbituric acid reactive substances (TBARS), TNF-α, Fas, caspase-8 and caspase-3 levels, while significantly increasing the glutathione/oxidized glutathione (GSH/GSSG) ratio and Bcl-2. Pae treatment alone led to increased Nrf2 nuclear accumulation, caused a slight increase in phosphorylated PI3K/Akt, restored ISO-induced Keap1 elevation, and upregulated HO-1, NQO1, and glutathione S-transferase (GST) activities [113]. This suggests that Nrf2/HO-1 signaling and PI3K/Akt activation may be involved in the protective effect of Pae on myocardium.

Protocatechuic acid (PCA) is a natural phenolic compound, which exists in green tea, vegetables, fruits and various plants. It can improve cardiac dysfunction, cardiomyocyte loss, fibrosis and inflammatory response, and reduce myocardial damage markers (CK-MB, LDH and cTnT), MDA, TNF-α, IL-1β and NF-κB expression, increase GSH and its derived enzymes, and also inhibited Bax, caspase-3, TGF-β1 and MMP-9 expression. PCA enhances the cellular antioxidant defense system by increasing the expression of Nrf2 and HO-1 [114]. Therefore, PCA can be used as an alternative therapeutic drug to improve the molecular, biochemical and histological changes caused by MI.

Resveratrol (RE) belongs to polyphenolic phytoalexin, which has been widely studied due to its antioxidant, anti-inflammatory, pro-angiogenic and other biological activities [160]. RE can mediate the inhibition of mTOR/TTP/NLRP3 mRNA signal by activating AMPK, and also improve the expression of antioxidant molecules by activating Nrf2 [115]. Another study also found that Resveratrol improved the MI size of MIRI rats, decreased the levels of myeloperoxidase (MPO), MDA, CK and LDH, and significantly enhanced the activities of SOD and GSH-Px. Under the stimulation of I/R, the dissociation of Nrf2 from Keap1 increased and was translocated to the nucleus, showing a significant increase in the protein expression of Nrf2 and HO-1, which was further increased after RE intervention [116]. Existing studies have shown that Nrf2 signaling plays an important role in RE protecting the heart from injury. However, further identification of other molecules and mechanisms involved in cardioprotection and elucidation of potential crosstalk between upstream and downstream signaling molecules are needed.

Polydatin (PD), an important glucoside of RE, is widely distributed in many plants [161] and can treat various diseases related to oxidative stress, inflammation and apoptosis [162]. PD provides myocardial protection against apoptosis and ROS production in acute MI. However, Nrf2 knockdown significantly reversed the effect of PD, upregulated caspase-3 and Bax expression, and suppressed Bcl-2 and HO-1 expression [117].

### 4.4. Polysaccharides and Glycosides

Catalpol belongs to iridoid glucoside and is the main active ingredient extracted from Rehmanniae Radix, which has the effects of antioxidation, promoting angiogenesis and relieving inflammatory pain [163,164]. In vivo and in vitro studies showed that catalpol can reduce the content of inflammatory markers and oxidative stress by regulating the Nrf2/HO-1 signaling pathway, improving MIRI and protecting cardiomyocyte injury model induced by OGD/R [118].

Astragaloside IV (ASI), a triterpene glycoside extracted from Astragalus membranaceus, has been shown to have potential protective effects against cardiovascular diseases [165]. It promotes the expression of Nrf2 in the nucleus of H9c2 cells and activates Nrf2 downstream gene promoters (NQO1, SOD2 and Txn-1), attenuating oxidative stress in cardiomyocytes [119]. It also improved H/R-induced myocardial injury, increased cardiomyocyte viability and SOD expression, and decreased ROS levels as well as content of LDH, MDA, IL-6 and TNF-α. This study also confirmed that ASI regulated the protein expression of Nrf2, HO-1 and BTB domain and CNC homolog 1 (Bach1) in the nucleus [120]. Interestingly, PI3K inhibitors could partially counteract the above effects, suggesting that PI3K/Akt/HO-1 signaling pathway may be a potential target of ASI against MIRI.

Crocin, also known as crocin A, is a water-soluble carotenoid isolated from the stigma of *Crocus sativus* L., which has cardioprotective activity [166]. There is experimental evidence that saffron not only alleviates I/R-induced left ventricular dysfunction and MI size, but also reduces the levels of Bax, caspase 3, miR-34a and endoplasmic reticulum stress biomarkers (GRP78 and CHOP), while increasing the levels of Bcl-2, Sirt1, Nrf2 and HO-1. Notably, Sirt1 blocker inhibited the effect of crocin, and miR-34a negatively regulated the effect of crocin on Sirt1/Nrf2 signaling pathway [121]. This suggests that crocetin inhibits I/R-induced ER stress and cardiomyocyte apoptosis by downregulating miR-34a and activating Sirt1/Nrf2 pathway.

### 4.5. Steroids

Ruscogenin (RUS), a bioactive steroidal saponin derived from *Ophiopogon japonicus*, has been identified as a potential drug for the treatment of cardiac diseases [167]. In vitro and in vivo experiments showed that it significantly down regulated the expression of Keap1, ACSL4 and FTL, and increased the levels of Nrf2, HO-1 and GPX4 in MI mice and OGD injured H9c2 cardiomyocytes. Importantly, RUS treatment increased the expression of branched-chain amino acid transaminase 1 or 2 (BCAT1 and BCAT2) in patients with MI; conversely, BCAT1 or BCAT2 siRNA induced the degradation of Nrf2 and HO-1 in cardiomyocytes and promoted ferroptosis [122]. It can be seen that RUS may activate Keap1/Nrf2/HO-1 pathway by upregulating BCAT1 and BCAT2 to alleviate ferroptosis induced by MI, thereby exerting its cardioprotective effect.

Dioscin, a natural steroid saponin extracted from various plants, can activate Nrf2 and Sirt2 signaling pathways and affect the levels of its downstream target genes (HO-1, NQO1, GST, GCLM, Keap1 and FOXO3a) to prevent oxidative stress in H9c2 cells and alleviate DOX-induced cardiotoxicity [123]. In the coronary heart disease (CHD) model, 80 mg/kg dioscin also antagonized apoptosis, inflammation and oxidative stress by promoting Sirt1/Nrf2 and inhibiting p38 MAPK and PARP/p53 expression [168].

### 4.6. Alkaloids

Neferine (NEF), originating from the green embryo of the mature seeds of lotus (Nelumbo nucifera), can exert anti-inflammatory, anti-apoptotic and antioxidant biological effects by regulating a variety of signaling pathways. In vitro studies have demonstrated that NEF can reduce the content of MDA, increase the expression of SOD and CAT, and improve mitochondrial dysfunction in injured H9c2 cells, which is closely related to the up regulation of Sirt1 and Nrf2 expression [124]. Silencing Sirt1 reversed the above process, suggesting that the activation of Sirt1/Nrf2 signaling pathway is a key factor for NEF to improve I/R injury in H9c2 cells.

Stachydrine (STA), the main component purified from the leaves of the Chinese herb Leonurus heterophyllus, has a variety of pharmacological properties, especially for cardiovascular diseases [169]. A 50 µM STA treatment significantly decreased the expression of LDH, MDA, caspase-3 and the number of TUNEL positive cells and increased SOD activity in H9c2 cells induced by H/R. In addition, STA reduced the secretion of apoptotic cytokines and oxidative stress molecules produced by H/R stimulation by activating Sirt1 and Nrf2/HO-1 signaling pathways to improve MI related symptoms and inflammation [125].

### 4.7. Phenylpropane

Plantamajorside (PMS) is a phenylpropanoid glycoside extracted from plantain, which can be anti-inflammatory, antioxidant and anti-fibrotic [170]. Studies have shown that PMS can improve the viability of H9c2 cells and inhibit ROS, TNF-α, IL-6, IL-1 β, Bax and caspase 3 production, increased the expression of SOD, CAT, GSH-Px and Bcl-2 stimulated by H/R. Further studies revealed that PMS inhibited H/R-induced NF-κB activation, and activated the Akt/Nrf2/HO-1 signaling pathway, ultimately alleviating the inflammatory response, oxidative stress, and apoptosis in injured cardiomyocytes [126].

Schisandrin B (Sch B), extracted from the fruit of Schisandra chinensis, belongs to one of the dibenzocyclooctadiene derivatives and has shown biological activity in the treatment of cardiovascular diseases [171]. In terms of cardiac effects, Sch B exhibited cardioprotection in an Nrf2 dependent manner. HO-1, NQO1 were decreased, and Keap1 was upregulated in H9c2 cells induced by H/R, while Nrf2 nuclear aggregation was decreased, and Sch B intervention reversed this effect. Si-Nrf2 counteracts the protective mechanism of Sch B on the heart [127]. Notably, Si-AMPK significantly decreased the expression of Nrf2. This suggests that knockdown of AMPK can inhibit the transmission of nfr2 and its downstream signals. In conclusion, Sch B alleviates oxidative stress and inflammatory response after MIRI dependent on AMPK/Nrf2 signaling pathway.

### 4.8. Quinones

Plumbagin (PL) is a bioactive naphthoquinone isolated from the plant plumbago, which has a variety of pharmacological properties and biological benefits [172]. After the intervention of PL on MIRI, rats showed better antioxidant and anti-inflammatory status, decreased ROS generation, increased activities of antioxidant enzymes (GSH, SOD, CAT, GPX and GST) and inflammatory markers (NF-κB, COX-2 and iNOS) expression decreased. In addition, it also induces Nrf2 activation, accompanied by increased NQO1, GST and HO-1 protein expression, which in turn reduces MIRI [128].

Aloin, which has various pharmacological properties such as anti-cancer and anti-inflammatory properties, is a natural anthraquinone glycoside found in the leaves of aloe vera plants [173]. It has a protective effect on ISO-induced cardiac hypertrophy in rats, and part of its mechanism is to stabilize the redox system and resist myocardial fibrosis by upregulating the levels of Nrf2 and HO-1 [174]. Another study found that silencing Nrf2 counteracted aloin mediated ROS, LDH, MDA, TNF-α, IL-6 and IL-1β reduced as well as elevated activity of antioxidant stress kinase SOD which, in turn, aggravated the oxidative stress and inflammatory response of cardiomyocytes induced by simulated ischemia/reperfusion (SI/R) [129].

### 4.9. Others

Anethole is a kind of aromatic compound, which can resist ISO-induced cardiomyocyte injury. The potential mechanism mainly includes two pathways, one is to increase mitochondrial antioxidant enzyme activity by activating Nrf2/HO-1 pathway, and the other is to reduce TLR4/MYD88 pathway to improve myocardial inflammatory response and apoptosis [131].

Kinsenoside (KD) is the main bioactive component naturally isolated from *Anoectochilus roxburghii*, which has anti-inflammatory, anti-hyperglycemic, anti-hyperlipidemic and vascular protective effects [175]. By increasing AKT phosphorylation and Nrf2 translocation to the nucleus, it significantly upregulates the expression of SOD, GSH, HO-1 and GPX4, inhibits MDA accumulation, iron accumulation, Mito-ROS and COX2 production, thereby reducing mitochondrial dysfunction in myocardial I/R and increasing antioxidant function [176].

Similarly, total flavonoids from *C. chinese* (benth.) O. Ktze (TFCC), as a traditional Chinese herbal medicine, is also mediated by blocking oxidative stress. TFCC treatment prevented hypoxia/reoxygenation (A/R)-induced apoptosis in H9c2 cells and low expression of AKT phosphorylation, increasing nuclear translocation of Nrf2 and expression of HO-1 [132]. The above process was blocked by AKT inhibitor, which indicated that TFCC-induced Nrf2/HO-1 activation and cytoprotection may be completed by promoting the phosphorylation of AKT.

*Ginkgo biloba* extract-761 (EGb 761), which is composed of Ginkgo flavonoids and terpene lactones, has been widely used to treat various cardiovascular and cerebrovascular diseases [177]. Studies have revealed a variety of mechanisms of action of EGb, including improving the degree of necrosis, edema and inflammatory infiltration of cardiomyocytes, and inhibiting the expression of proinflammatory cytokines (TNF-α, IL-6 and IL-1 β), decreasing the expression of apoptotic factors (caspase3 and Bax), and enhanced the activity of antioxidant enzymes (SOD and GSH-Px) in myocardial tissue [133]. Among them, the effect of EGb against oxidative stress is to first activate Akt phosphorylation, promote Nrf2 to translocate into the nucleus and upregulate HO-1 expression, so Akt/Nrf2/HO-1 pathway seems to become an important target to protect MIRI in rats [133].

In summary, different natural products can protect the heart by regulating Nrf2/HO-1, AMPK, PI3K/Akt, NF-κB, Sirt, ERK, JNK and other related signals, such as enhancing cardiac remodeling, improving MI, alleviating MI, and protecting I/R-induced myocardial apoptosis (Table 2). This important function implies its potential role in participating in cardiovascular diseases. However, the molecular crosstalk mechanism between natural products and signaling pathways is not fully understood, and the specific effects of natural compounds in various pathological conditions of the heart need to be further evaluated.

## 5. Conclusions and Prospects

**Figure 3:**
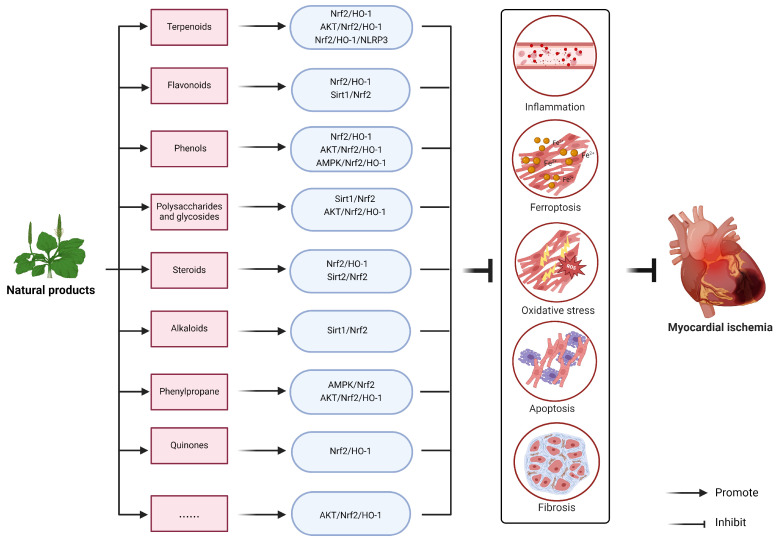
The effects of natural products targeting Nrf2-related signaling pathway on myocardial ischemia. The natural product inhibits pathological mechanisms such as inflammatory response, ferroptosis, oxidative stress, apoptosis and fibrosis in cardiomyocytes through the Nrf2/HO-1-related signaling pathway, thereby alleviating myocardial ischemic injury. (Created with BioRender.com, accessed on 10 March 2024).

As an important pathway of anti-oxidative stress, Nrf2 signaling is involved in alleviating the pathological changes in myocardial ischemia. A large number of studies have shown that many NPs can effectively and safely relieve myocardial ischemia injury through Nrf2 signaling. With the deepening of research, Nrf2-based signaling pathways have gradually emerged, which are often associated with HO-1, AMPK, PI3K/Akt, NF-κB, Sirt1, ERK and other signaling pathways interact and influence each other, forming a complex pharmacological network for NPs to protect the heart (Figure 3).

Although the evidence of the cardioprotective effect of NPs is relatively clear, the mechanism of Nrf2 as a driver needs further verification. At present, the research on drugs targeting Nrf2 in the treatment of ischemic heart disease is mostly based on the level of cells and animal models, lacking factual evidence of clinical research. NPs are helpful in both the development and prevention of cardiovascular events. To truly translate basic experimental results into clinical applications, dose-specific clinical trials are still required to determine the anti-inflammatory and antioxidant therapeutic potential of natural compounds, as well as their potential toxicity and safety over the long term. Meanwhile, the comparison of the mechanism of action of natural drugs with different structures and types to protect the heart should be clarified, and its specific effect should be clarified. Next, an in-depth exploration of the regulatory network of NPs targeting Nrf2 to protect the heart will help delay the development of cardiovascular disease and improve its prognosis, which may be a promising therapeutic strategy.

## Figures and Tables

**Table 1:** Experimental model and conclusion of natural products improving myocardial ischemic injury.

Category	Natural Product	Model	Route of Medication	Research Type	Conclusions	References
Terpenoids	Andrographolide	C57BL/6 mice, *N* = 110, H9c2 cell	Orally/Cell culture	Preclinical	Andr alleviates adverse cardiac remodeling following myocardial infarction through enhancing Nrf2 signaling pathway	[91]
	Panaxatriol saponin	SD rats *N* = 60, H9c2 cell	Intragastric administration/Cell culture	Preclinical	PTS has therapeutic potential for MIRI by targeting Keap1/Nrf2 activity	[92]
	Ginsenoside Rd	SD rats *N* = 24	Administrated intraperitoneally	Preclinical	GsRd protects against myocardial I/R injury via Nrf2/HO-1 signaling	[93]
	Ginsenoside Rh2	SD rats *N* = 40, Neonatal rat cardiomyocytes	Intragastric administration/Cell culture	Preclinical	GRh2 could reduce oxidative stress and inflammation in cardiomyocytes after reperfusion	[94]
	Ginsenoside Rb2	H9c2 cell	Cell culture	Preclinical	The underlying mechanism of ginsenoside Rb2 in H9c2 cells could be standardized to Nrf2/HO-1 signaling pathway, inhibiting cell apoptosis and regaining cell proliferation	[95]
	Triptolide	Wistar rats *N* = /	Administrated intraperitoneally	Preclinical	A novel cardioprotective effect of TPL in rats with I/R injuries, wherein the activation of Nrf2/HO-1 signaling was involved	[96]
	Betulinic acid	H9c2 cell	Cell culture	Preclinical	BA protects the H9c2 cells from I/RI by inhibiting oxidative stress and cell apoptosis. The cardio-protective effects were mediated by the Nrf2/HO-1, p38 and JNK pathways	[97]
	Maslinic acid	SD rats *N* = 60, H9c2 cell	Intraperitoneal injection	Preclinical	MA exerts its cardioprotective effect through regulating the crosstalk between the Nrf2 and NF-κB pathways	[98]
	Glaucocalyxin A	H9c2 cell	Cell culture	Preclinical	GLA protected H9c2 cells from H/R-stimulated oxidative damage, which was mediated by the Akt/Nrf2/HO-1 signaling pathway	[99]
	Costunolide	C57BL/6 mice *N* = 64, H9c2 cell	Gavage/Cell culture	Preclinical	Activation of Nrf2/Keap1 using Cos may be a therapeutic strategy for myocardial I/R injury	[100]
	Lutein	SD rats *N* = 40	Orally	Preclinical	LU exhibits potent cardioprotective activity against ISO-induced cardiotoxicity	[101]
Flavonoids	Hesperetin	Mice *N* = 50	Intragastrically	Preclinical	HESP plays a protective role in ISO-induced myocardial ischemia by modulating oxidative stress, inflammation, and apoptosis via Sirt1/Nrf2 pathway activation	[102]
	Baicalin	H9c2 cell	Cell culture	Preclinical	BI cardiomyocytes H9c2 apoptosis aroused by hypoxia might be achieved through activating Nrf2/HO-1-mediated HIF1α/BNIP3 pathway	[103]
	Pinocembrin	SD rats *N* = 56, H9c2 cell	Injected intravenously through the tail vein/Cell culture	Preclinical	PCB ameliorated cardiac functions and remodeling resulted from PIHF by ROS scavenging and Nrf2/HO-1 pathway activation which further attenuated collagen fibers deposition and apoptosis, and facilitated angiogenesis	[104,105]
	Icariin	H9c2 cell	Cell culture	Preclinical	ICA attenuates H/R-induced ferroptosis of cardiomyocytes by activating the Nrf2/HO-1 signaling pathway	[106]
	Wogonoside	C57BL/6 mice *N* = 35	Intraperitoneally injected	Preclinical	WG exerted the protective role against I/R-induced myocardial injury by suppression of apoptosis, inflammation, and fibrosis via activating Nrf2/HO-1 pathway	[107]
	Visnagin	Wistar rats *N* = 36	Orally	Preclinical	Activation of Nrf2/HO-1 signaling and PPARγ mediates the cardioprotective effect of VIS	[108]
	Isoliquiritigenin	C57BL/6 mice *N* = 50	Intraperitoneal injection	Preclinical	Activation of Nrf2/HO-1 pathway has an essential role in ISL-induced cardiac protection by alleviating myocardial oxidative stress and inflammation response in mice with AMI	[109]
Phenols	Salvianolic acid B	SD rats *N* = 108	Intraperitoneal injection	Preclinical	Sal B contributed to protecting MI by inhibiting ferroptosis via activating the Nrf2 signaling pathway	[110]
	Lithospermic acid	C57BL/6 mice *N* = 50, H9c2 cell	Orally/Cell culture	Preclinical	LA protects against MI/R-induced cardiac injury by promoting eNOS and Nrf2/HO-1 signaling via phosphorylation of AMPKα	[111]
	Kazinol B	H9c2 cell	Cell culture	Preclinical	KB prevented H/R-induced cardiomyocyte injury via modulating the AKT and AMPK-mediated Nrf2 induction	[112]
	Paeonol	SD rats *N* = 40	Subcutaneous injection	Preclinical	Pae exerts significant cardioprotective effects against ISO-induced myocardial infarction in rats	[113]
	Protocatechuic acid	Wistar rats *N* = 35	Orally	Preclinical	PCA as an alternative therapeutic agent to attenuate the molecular, biochemical, and histological alterations associated with MI development	[114]
	Resveratrol	SD rats *N* = 24	Orogastric gavaged	Preclinical	Myocardial protective mechanism of RE during CIH and suggest that resveratrol treatment may be useful to counteract OSA-associated cardiac injury	[115,116]
	Polydatin	SD rats *N* = /, H9c2 cell	Intraperitoneal injection/Cell culture	Preclinical	PD effectively inhibited hypoxia- and AMI-induced myocardial damage by promotion of Nrf2/HO-1 signaling	[117]
Polysaccharides and glycosides	Catalpol	C57BL/6 mice *N* = 30, Human cardiomyocytes AC16 cells	Intraperitoneal injection/Cell culture	Preclinical	Catalpol exerted significant cardioprotective effects following myocardial ischemia, possibly through the activation of the Nrf2/HO-1 signaling pathway	[118]
	Astragaloside IV	SD rats *N* = 45, H9c2 cell	Orally/Cell culture	Preclinical	ASI prevented heart failure by counteracting oxidative stress through the Nrf2/HO-1 pathway	[119,120]
	Crocin	C57BL6/J mice *N* = 32, Neonatal mouse cardiomyocytes (NMCMs)	Orally/Cell culture	Preclinical	Crocin attenuates I/R-induced cardiomyocyte apoptosis via suppressing ER stress, which is regulated by the miR-34a/Sirt1/Nrf2 pathway	[121]
Steroids	Ruscogenin	ICR mice *N* = 72, H9c2 cell	Intraperitoneal injection/Cell culture	Preclinical	BCAT1/BCAT2 could alleviate MI-induced ferroptosis through the activation of the Keap1/Nrf2/HO-1 pathway and RUS exerted cardioprotective effects via BCAT1/BCAT2	[122]
	Dioscin	SD rats *N* = 50, H9c2 cell	Intraperitoneal injection/Cell culture	Preclinical	Dioscin alleviated DOX-induced cardiotoxicity through modulating miR-140-5p-mediated myocardial oxidative stress	[123]
Alkaloids	Neferine	H9c2 cell	Cell culture	Preclinical	NEF preconditioning attenuated H/R-induced cardiac damage via suppressing apoptosis, oxidative stress, and mitochondrial dysfunction, which may be partially ascribed to the activation of Sirt1/Nrf2 signaling pathway	[124]
	Stachydrine	H9c2 cell	Cell culture	Preclinical	STA protects H/R injury and inhibits oxidative stress and apoptosis in cardiomyocytes by activation of the Sirt1-Nrf2 pathway	[125]
Phenylpropane	Plantamajoside	H9c2 cell	Cell culture	Preclinical	PMS protected against myocardial I/R injury via attenuating oxidative stress, inflammatory response and apoptosis	[126]
	Schizandrin B	H9c2 cell	Cell culture	Preclinical	Sch B exerts cardioprotection on H/R injury in H9c2 cells due to its antioxidant and anti-inflammatory activities via activation of the AMPK/Nrf2 pathway	[127]
Quinones	Plumbagin	C57BL6/J mice *N* = 40	Intraperitoneal injection	Preclinical	Protective role of PL against myocardial I/R injury by regulating antioxidant and inflammatory mechanisms	[128]
	Aloin	H9c2 cell	Cell culture	Preclinical	Aloin may antagonize SI/R-induced cardiomyocyte injury by attenuating excessive oxidative stress and inflammation	[129]
Others	Kinsenoside	C57BL6/J mice *N* = 88	Orally	Preclinical	KD may exert anti-ferroptosis effect in myocardial I/R injury by decreasing mitochondrial dysfunction and increasing anti-oxidation through the Akt/Nrf2/HO-1 signaling pathway	[130]
	Anethole	Wistar rats *N* = 30	Gastric lavage	Preclinical	Anethole may retain a cardio-protective potential by controlling myocardial oxidative stress (through Nrf2 pathway) and diminishing inflammation and apoptosis via the TLR4/MYD88 pathway	[131]
	TFCC *Clinopodium chinense* (Benth.) O. Ktze	SD rats *N* = 120, H9c2 cell	Dosed intragastrically/Cell culture	Preclinical	TFCC protects against myocardial injury and enhances cellular antioxidant defense capacity by inducing the phosphorylation of AKT, which subsequently activated the Nrf2/HO-1 signaling pathway	[132]
	*Ginkgo biloba* extract-761	SD rats *N* = 40	Gavage	Preclinical	EGb 761 might inhibit the apoptosis of myocardial cells and protect the myocardium by activating the Akt/Nrf2 pathway, increasing the expression of HO-1, decreasing oxidative stress and repressing inflammatory reaction	[133]

**Table 2:** The mechanism of natural products improving myocardial ischemic injury.

Category	Molecular Formula	Model	Related Gene/Cytokines/Protein	Pathway	References
Terpenoids					
Andrographolide	C_20_H_30_O_5_	C57BL/6 mice, H9c2 cell	↑: SOD2, NQO1, GPX, Nrf2, HO-1 ↓: TNF-α, IL-1β, IL-6, MCP-1, p-IκBα, p-p65, p67 phox, Gp91, NOX4, TGF-β, p-smad3	NF-κB, Nrf2/HO-1	[91]
Panaxatriol saponin	C_30_H_52_O_4_	SD rats, H9c2 cell	↑: SOD1, SOD2, HO-1 ↓: cleaved caspase-3, cleaved PARP-1, Bax, Cyt-c, MB, cTn-T, CK, LDH	Keap1/Nrf2/HO-1	[92]
Ginsenoside Rd	C_48_H_82_O_18_	SD rats	↑: Nrf2, HO-1 ↓: CK, LDH, cTnI	Nrf2/HO-1	[93]
Ginsenoside Rh2	C_36_H_62_O_8_	SD rats, Neonatal rat cardiomyocytes	↑: Nrf2, HO-1, SOD, GSH-Px ↓: IL-1β, IL-18, TNF-α, NLRP3, ASC, caspase-1, MDA, LDH, CK, CK-MB	Nrf2/HO-1/NLRP3	[94]
Ginsenoside Rb2	C_53_H_90_O_22_	H9c2 cell	↑: Nrf2, HO-1 ↓: CK-MB, cTn-1, LDH	Nrf2/HO-1	[95]
Triptolide	C_20_H_24_O_6_	Wistar rats	↑: Nrf2, HO-1, GPX, GSH, SOD ↓: TNF-α, IL-1β, IL-6, MDA	Nrf2/HO-1	[96]
Betulinic acid	C_30_H_48_O_3_	H9c2 cell	↑: Nrf2, HO-1, Bcl-2 ↓: LDH, caspase-3, Bax, p38, JNK	Nrf2/HO-1, p38, JNK	[97]
Maslinic acid	C_30_H_48_O_4_	SD rats, H9c2 cell	↑: GSH, SOD, p-Nrf2, HO-1, NQO1 ↓: LDH, CK-MB, MAD, Keap-1, p-IκBα, p-P65, TNF-α	Nrf2/HO-1, NF-κB	[98]
Glaucocalyxin A	C_20_H_28_O_4_	H9c2 cell	↑: SOD, GSH-Px, Bcl-2, p-Akt, Nrf2, HO-1 ↓: Bax, caspase-3	Akt/Nrf2/HO-1	[99]
Costunolide	C_15_H_20_O_2_	C57BL/6 mice, H9c2 cell	↑: Bcl-2, NQO1, HO-1, Nrf2 ↓: cleaved caspase-3, Bax, MDA, SOD	Keap1/Nrf2	[100]
Lutein	C_40_H_56_O_2_	SD rats	↑: CAT, SOD, Nrf2, HO-1 ↓: MDA, cTnT, CK-MB, LDH, IL-1β, TNF-α, NF-κB p65, caspase-3, caspase-9	Nrf2/HO-1	[101]
Flavonoids					
Hesperetin	C_16_H_14_O_6_	mice	↑: CAT, SOD, GSH, Bcl-2, Sirt1, Nrf2, NQO1, HO-1 ↓: CK, LDH, MDA, IL-6, TNF-α, Bax, caspase-3	Sirt1/Nrf2	[102]
Baicalin	C_21_H_18_O_11_	H9c2 cell	↑: Bcl-2, HIF1α, BNIP3, Nrf2, HO-1 ↓: p53, Bax, cleaved-caspase 9, cleaved-caspase 3	HIF1α/BNIP3, Nrf2/HO-1	[103]
Pinocembrin	C_15_H_12_O_4_	SD rats, H9c2 cell	↑: Bcl-2, SOD, Nrf2, HO-1 ↓: p53, Bax, cleaved-caspase 3, MDA	Nrf2/HO-1	[104,105]
Icariin	C_33_H_40_O_15_	H9c2 cell	↑: Nrf2, HO-1, GPX4, SOD, CAT ↓: LDH, ACSL4, MDA	Nrf2/HO-1	[106]
Wogonoside	C_22_H_20_O_11_	C57BL/6 mice	↑: Bcl-2, Nrf2, HO-1, NQO1 ↓: Mb, CK-MB, cTnI, cleaved caspase-3, cleaved caspase-9, Bax, TNF-α, IL-6, iNOS, α-SMA, TGF-β	Nrf2/HO-1	[107]
Visnagin	C_13_H_10_O_4_	Wistar rats	↑: Nrf2, HO-1, Bcl-2, PPARγ, GSH, SOD, CAT, GPX ↓: MDA, NF-κB p65, Bax, caspase 3, caspase 9, CTnI, CK-MB, LDH, TNF-α, IL-6	Nrf2/HO-1	[108]
Isoliquiritigenin	C_15_H_12_O_4_	C57BL/6 mice	↑: GSH-Px, SOD, Nrf2, HO-1 ↓: MDA, p-p65, IL-6, IL-1β, TNF-α, MIP1α, MIP2, p-IKKα/β, p-P65, p-IκBα	Nrf2/HO-1	[109]
Phenols					
Salvianolic acid B	C_36_H_30_O_16_	SD rats	↑: Nrf2, HO-1, GSH, xCT, GPX4, Fth1, Fpn1 ↓: MDA, CK, CK-MB, LDH	Nrf2/HO-1	[110]
Lithospermic acid	C_27_H_22_O_12_	C57BL/6 mice, H9c2 cell	↑: eNOS, Nrf2, GPX, SOD2, NQO1, Bcl-2 ↓: TnT, CK-MB, ROS, GP91, NOX4, p67 phox, p47 phox, Caspase-3, Bax	AMPK, Nrf2/HO-1	[111]
Kazinol B	C_25_H_28_O_4_	H9c2 cell	↑: Bcl-2/Bax, ATP, GSH-Px, SOD, Nrf2, HO-1 ↓: caspase-3, cleaved PARP, MDA, LDH, p-AKT, p-AMPKα	AKT, AMPK, Nrf2/ARE/HO-1	[112]
Paeonol	C_9_H_10_O_3_	SD rats	↑: GSH/GSSG, Bcl-2, Nrf2, HO-1, NQO1, GST, p-PI3K, p-Akt ↓: Bax, TBARS, TNF-α, Fas, caspase-8, caspases-3	Nrf2/HO-1, PI3K/Akt	[113]
Protocatechuic acid	C_7_H_6_O_4_	Wistar rats	↑: CAT, SOD, GSH, GPX, GR, Nrf2, HO-1, Bcl-2, TIMP-3 ↓: CK, LDH, CTnT, MDA, NO, TNF-α, IL-1β, Bax, caspase-3, TGF-β1, MMP-9	Nrf2/HO-1	[114]
Resveratrol	C_14_H_12_O_3_	SD rats	↑: SOD, GSH, GSH-PX, p-mTOR/mTOR, SOD2, Nrf2, HO-1, p-AMPK/AMPK ↓: CK, LDH, MDA, NOX2, NOX4, p-IRE/IRE, P-PERK/PERK, GRP78, NLRP3, caspase-1, IL-1β, MPO	AMPK, Nrf2	[115,116]
Polydatin	C_20_H_22_O_8_	SD rats, H9c2 cell	↑: Nrf2, HO-1, Bcl-2 ↓: caspase-3, Bax	Nrf2/HO-1	[117]
Polysaccharides and glycosides					
Catalpol	C_15_H_22_O_10_	C57BL/6 mice, Human cardiomyocytes AC16 cells	↑: SOD2, GSH, Bax, Nrf2, HO-1 ↓: cTnI, CK-MB, NPPB, BNP, MDA, ROS, IL-1β, IL-6, IL-8, TNF-α, cleaved caspase 3	Nrf2/HO-1	[118]
Astragaloside IV	C_41_H_68_O_14_	SD rats, H9c2 cell	↑: CAT, GSH, SOD, Nrf2, HO-1, NQO1, SOD2, Txn-1, PI3K, p-Akt ↓: CK, Keap-1, LDH, MDA, IL-6, TNF-α, Bach1	PI3K/Akt/Nrf2/HO-1	[119,120]
Crocin	C_44_H_64_O_24_	C57BL6/J mice, Neonatal mouse cardiomyocytes (NMCMs)	↑: Bcl-2, Sirt1, Nrf2, HO-1 ↓: miR-34a, GRP78, CHOP	miR-34a/Sirt1/Nrf2	[121]
Steroids					
Ruscogenin	C_27_H_42_O_4_	ICR mice, H9c2 cell	↑: GPX4, SOD, GSH, BCAT1, BCAT2, Nrf2, HO-1 ↓: ACSL4, FTL, MDA, Keap1	Keap1/Nrf2/HO-1	[122]
Dioscin	C_45_H_72_O_16_	SD rats, H9c2 cell	↑: SOD, GSH, GSH-Px, Sirt2, Nrf2, HO-1, NQO1, GST, GCLM, Sirt2, FOXO3a ↓: CK, LDH, MDA, miR-140-5p, Keap1	miR-140-5p, Nrf2, Sirt2	[123]
Alkaloids					
Neferine	C_38_H_44_N_2_O_6_	H9c2 cell	↑: Sirt1, Nrf2, SOD, CAT ↓: MDA, LDH	Sirt1/Nrf2	[124]
Stachydrine	C_7_H_13_NO_2_	H9c2 cell	↑: SOD, Sirt1, Nrf2, HO-1 ↓: LDH, MDA, caspase-3	Sirt1/Nrf2	[125]
Phenylpropane					
Plantamajoside	C_29_H_36_O_16_	H9c2 cell	↑: SOD, CAT, GSH-Px, Bcl-2, p-Akt, Nrf2, HO-1 ↓: TNF-α, IL-6, IL-1β, Bax, caspase 3, p-IκBα, p-P65	Akt/Nrf2/HO-1, NF-κB	[126]
Schizandrin B	C_23_H_28_O_6_	H9c2 cell	↑: SOD, GSH, Nrf2, NAD(P)H, NQO1, HO-1, p-AMPK ↓: LDH, MDA, TNF-α, IL-6, IL-1β, IL-8, TGF-β, IL-10	AMPK/Nrf2	[127]
Quinones					
Plumbagin	C_11_H_8_O_3_	C57BL6/J mice	↑: GSH, SOD, CAT, GPX, GST, Nrf2, HO-1, NQO1 ↓: NF-κB, COX-2, iNOS, MCP-1, IL-6, IL-8, TNF-α	Nrf2	[128]
Aloin	C_21_H_22_O_9_	H9c2 cell	↑: SOD, Nrf2, HO-1 ↓: LDH, MDA, IL-6, IL-1β, TNF-α	Nrf2/HO-1	[129]
Others					
Kinsenoside	C_10_H_16_O_8_	C57BL6/J mice	↑: p-Akt, Nrf2, HO-1, SOD, GSH, HO-1, GPX4 ↓: MDA, CK-MB, COX2, ACSL4, Keap1	Akt/Nrf2/HO-1	[130]
Anethole	C_10_H_12_O	Wistar rats	↑: Nrf2, HO-1, SOD, CAT, GPX, GSH, GST, Bcl-2 ↓: Keap-1, CKMB, CK, CTnT, TNF-α, IL-1β, IL-6, NF-κB, Bax, caspase-3, caspase-9, TLR4, MYD88	Nrf2/HO-1, TLR4/MYD88	[131]
TFCC *Clinopodium chinense* (Benth.) O. Ktze	/	SD rats, H9c2 cell	↑: GSH-Px, CAT, SOD, POD, HO-1, Nrf2, HO-1, AKT, Bcl-2 ↓: cleaved caspase-3, caspase 9, Bax	AKT/Nrf2/HO-1	[132]
*Ginkgo biloba* extract-761	/	SD rats	↑: Bcl-2, p-Akt, HO-1, Nrf2 ↓: CK-MB, LDH, TnT, TNF-α, IL-6, IL-1β, Caspase-3, Bax	Akt/Nrf2	[133]

↑: up regulation; ↓: down regulation.

## Data Availability

The data that support the finding of this study are available from the corresponding author upon reasonable request.
